# Meaningful Human Control over AI for Health? A Review

**DOI:** 10.1136/jme-2023-109095

**Published:** 2023-09-20

**Authors:** Eva Maria Hille, Patrik Hummel, Matthias Braun

**Affiliations:** 1 Chair of Social Ethics & Ethics of Technology, Rheinische Friedrich-Wilhelms-Universität Bonn, Bonn, Germany; 2 Department of Industrial Engineering and Innovation Sciences, Eindhoven University of Technology, Eindhoven, The Netherlands

**Keywords:** Decision-making, Ethics

## Abstract

Artificial intelligence is currently changing many areas of society. Especially in health, where critical decisions are made, questions of control must be renegotiated: who is in control when an automated system makes clinically relevant decisions? Increasingly, the concept of meaningful human control (MHC) is being invoked for this purpose. However, it is unclear exactly how this concept is to be understood in health. Through a systematic review, we present the current state of the concept of MHC in health. The results show that there is not yet a robust MHC concept for health. We propose a broader understanding of MHC along three strands of action: enabling, exercising and evaluating control. Taking into account these strands of action and the established rules and processes in the different health sectors, the MHC concept needs to be further developed to avoid falling into two gaps, which we have described as theoretical and labelling gaps.

## Introduction

 The use of automated systems is playing an increasing role in various areas of society. Approaches such as machine learning, deep learning and artificial neural networks are shaping data processing and analysis and are used to build predictions in the areas of health, defence, transport, logistics, finance and others. In all these application areas, automated systems are able to accomplish their tasks without human control or intervention.^1^

However, a central task in dealing with automated systems is to ensure that they operate according to predefined goals, that the initial situation has been adequately mapped and that there are effective opportunities for intervention in the event of a possible failure of the system.

In order to be able to deal with these challenges conceptually, the concept of so-called ‘meaningful human control’ (MHC) is introduced. MHC encompasses the idea that ‘[…] humans not computers and their algorithms should ultimately remain in control of, and thus morally responsible for, relevant decisions […]’.^1^ The concept of MHC has its origins in debates about the use of automated systems in the context of defence and warfare. As Filippo Santoni de Sio and Jeroen van den Hoven^1^ have pointed out, the idea of MHC is guided by three central notions: first, keeping humans in the loop is not enough since it does not say much about the extent to which control is actually exercised and about the kinds of options and information available to human actors to make assessments. Second, merely ensuring substantive causal contributions of humans over automated processes is not enough either because of, for example, potentially imperfect psychological capacity to grasp and respond to the system’s capacity and behaviour. And third, simple forms of causal control might not be enough to ascribe the kinds of moral or legal responsibility that require stricter control conditions.

In health, there are various additional challenges to control automated systems used in different settings: for diagnostics and treatment recommendations^2 3^ (sometimes using artificial intelligence (AI)-driven decision support systems),^4^ in surgery and care (through the application of surgical and care robots),^5 6^ in telemedicine,^7^ in public health (eg, for pandemic monitoring),^8^ for triage decisions^9^ and many others. In all these areas, the first challenge to control is that decision-making is complex in medical contexts, where many different actors have to act with often very uncertain knowledge of the possible consequences and side effects. Moreover, in medical contexts, failure to act is often associated with severe consequences for the person concerned. Second, there are clearly negotiated moral and legal principles and rules within the framework of medicine, which require a high level of evidence as a basis for decision-making. Third, and this is particularly important for the conceptual definition of control, there are not only different actors involved in the application of automated systems in medicine, but also very diverse individual moral intuitions, some of which allow very different conclusions to be drawn about the required form of control. Unlike the context of autonomous weapons, individual preferences are of fundamental importance in medicine, for example, regarding what individual self-determination should encompass in clinical decision-making.

Our article examines and analyses the concept of MHC in the context of health. We show that the requirement of control, the actors needed for the implementation of control as well as the possibility of evaluating control over automated systems vary significantly. The findings offer important results for current and future debates on how we understand control when dealing with automated systems, who needs to exercise control over such systems in health contexts and how possible harm caused by automated systems could be mitigated.^10^

## Methods

**Figure 1:**
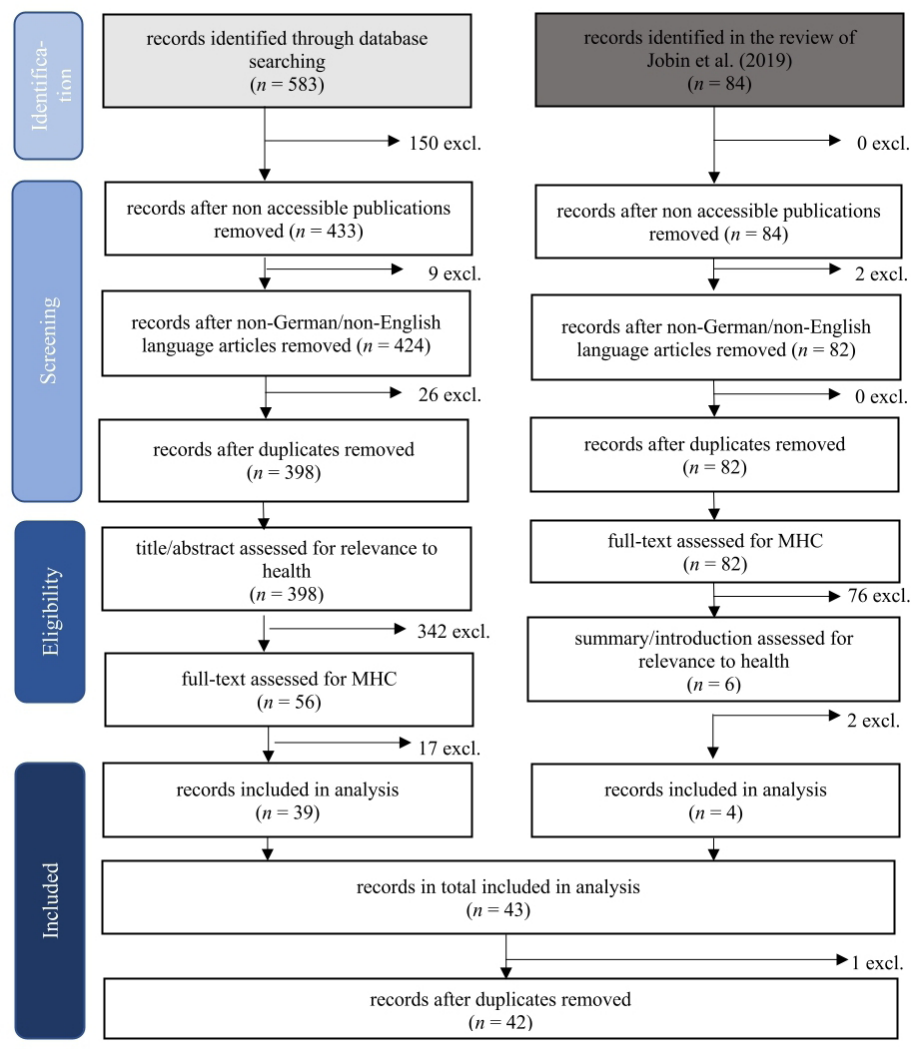
Flow chart of the review process based on the PRISMA framework. MHC, meaningful human control; PRISMA, Preferred Reporting Items for Systematic Reviews and Meta-Analyses.

We conducted a systematic review to investigate the current state of research and debate on the concept of MHC in the health sector. To gain a comprehensive understanding, we used two search strategies: first, a database search was conducted. Second, the dataset identified in the review of Anna Jobin and her colleagues^11^ was retrieved. Their review is one of the most comprehensive and up-to-date studies in the field of ethics guidelines for AI. It is thus an important resource for our research aim of providing an extensive overview of the current state of research on MHC in the health sector. Guided by the Preferred Reporting Items for Systematic Reviews and Meta-Analyses framework,^12^ we conducted both search strategies according to the four steps of identification, screening, eligibility and inclusion, which are presented below (figure 1).

As part of the first search strategy, we searched five different electronic databases (Google Scholar, PubMed, ScienceDirect, Scopus and Web of Science). Taking into account the Peer Review of Electronic Search Strategies for systematic reviews,^13^ we developed the following search strategy: (health* OR medic* OR clinic*) AND “meaningful human control”.^1^ We considered all literature published up to 27 September 2021, including ethics or policy guidelines. Five hundred eighty-three records were identified which created our dataset.

In a second step, we screened this dataset: the records had to be (1) accessible (either published open access or accessible via consortia of university libraries) and (2) published in English or German. All duplicates were removed. This resulted in a dataset of 398 records that went through the following eligibility process.

The eligibility process took place in two steps: first, we conducted a title and abstract assessment with regard to the health sector. Records were excluded if the title or abstract^2^ did not contain the terms “health*”, “medic*” or “clinic*” or if there was no other reference to a health context (through terms such as surgery or COVID-19/SARS-CoV-2).^3^ Through this step, we aimed to remove records in which a health-related term was only mentioned as an example and to keep those articles that actually dealt with a health-related issue. Second, we assessed the full text to see whether records actually mentioned the concept of MHC (either written out in full or abbreviated).^4^ Thirty-nine records were included in the subsequent analysis.

As part of the second search strategy, that is, the analysis of the dataset of Jobin and her colleagues,^11^ 84 records were included. During the entire screening process, we excluded two records due to a lack of accessibility. In the eligibility process of this search strategy, we first assessed the full text for MHC. Second, we checked the summaries or introductions of the remaining records in terms of their relevance to the health sector.^5^ Finally, three records were included in our review. Here, both search strategies were merged and checked for duplicates. After removing one duplicate, 42 records could be included in the following analysis (see the corresponding list in the online supplemental material).

The authors carried out a content analysis of 42 records in three cycles of coding by using the qualitative data analysis software Atlas.ti. At first, we marked the text paragraphs in which the term MHC was mentioned. If MHC was (part of) a subheading, all paragraphs of the subsection were considered.^6^ After several iterations of scanning the data, some relevant codes were discovered (such as tracing and tracking, the attribution of responsibility, different people who are involved and some others). During the first cycle coding, codes were set attributed in the previously marked paragraphs using inductive coding.^14^ After the first cycle coding, these codes were mapped using code mapping^14^ to identify code structures. The result of this process was a code scheme with nine code groups. For the three code groups *enablers*, *controllers* and *evaluators,* the codes assigned to the different code groups were added up in all three code groups to obtain a reasonably comparable result. In the second cycle coding, each paragraph was evaluated with respect to all nine code groups. The code *unspecified* was selected if no other code of the code group could be attributed or if the viewed paragraph did not specify, for example, who acts as an evaluator. All set codes were cross-checked by at least one other person to avoid individual cognitive bias. Finally, a consistency check was carried out between the different codes.

## Results

In our search, we found 42 health-related records that dealt with the concept of MHC. All records were published between the years 2016 and 2021. The fact that more than 80% of the records were published in 2019 or later indicates an increasing engagement with the concept of MHC in the health sector.

**Figure 2:**
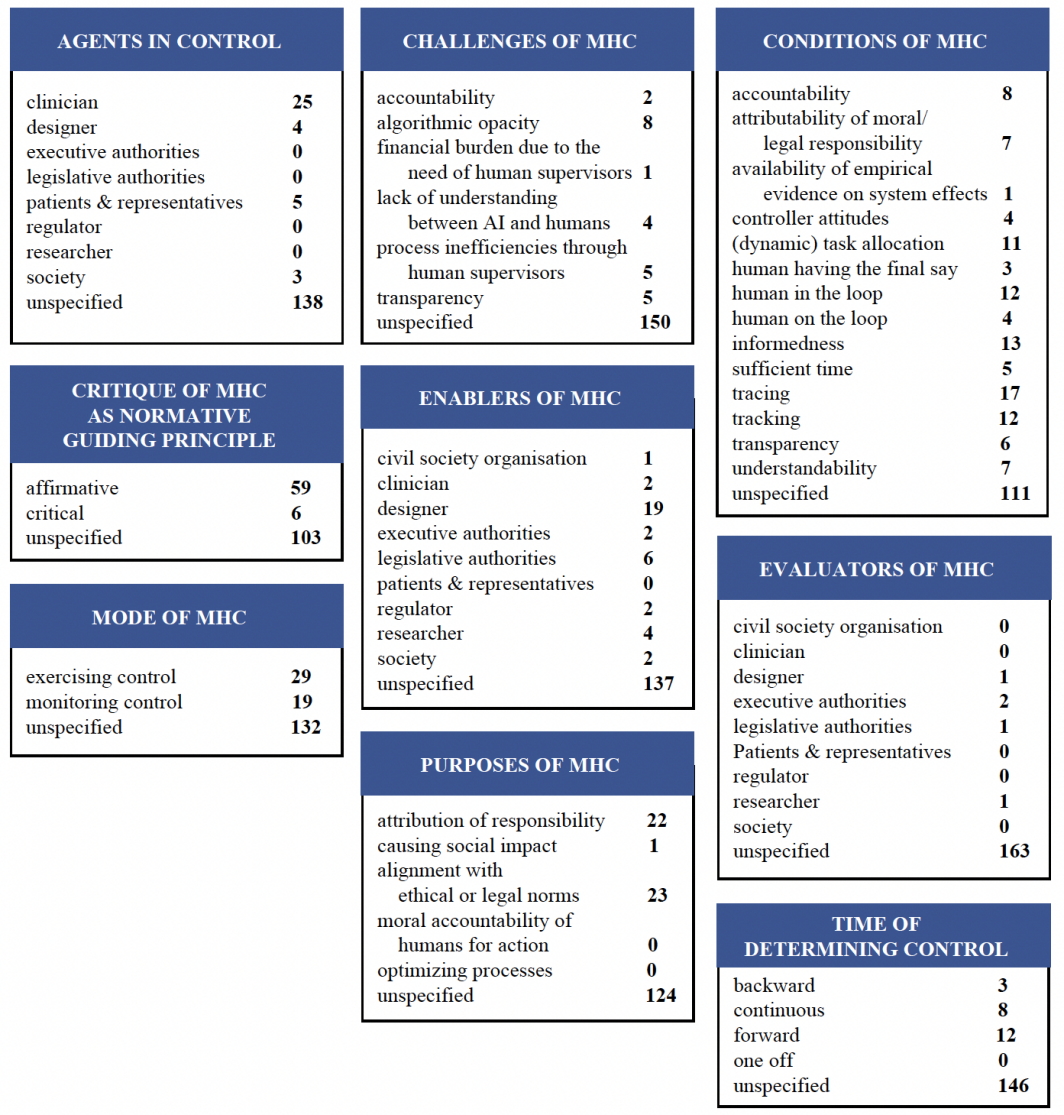
Results of the coding of the 42 records included. AI, artificial intelligence; MHC, meaningful human control.

We present our coding results (figure 2) by mapping the code groups to three action fields: (a) the enabling of MHC, (b) the action of controlling itself and (c) the evaluation of MHC. Within these three action fields, we present each code group based on its most frequent codes. In all code groups, the *unspecified* code occurs with high frequency (n=103–161, out of 168 text passages analysed).^7^ These figures show that many records mention MHC and take it to be a relevant concept when shaping AI in health, but do not explain what exactly they mean by MHC. This observation is underlined by the fact that 28 of the 42 records included contain only one or two paragraphs on MHC.

### Enabling control

First, we ask what is required to enable MHC when using automated systems in health settings. What purpose does MHC serve in these contexts? And who are those who (can) enable MHC? The different dimensions of what needs to be in place for MHC to be implemented within the use of automated systems in the health sector are indicated by the following code groups: *enablers*, *purposes of MHC* and *conditions of MHC*.

#### Enablers of MHC

Enablers are different human actors and institutions that aim to facilitate MHC in different health settings. They ensure that the necessary conditions for MHC are in place when AI-driven systems are used in the health sector. *Designers* in particular (n=19) occupy a decisive position here. By *designers* we mean all those who design, develop or programme AI. In the concept of MHC as discussed by Santoni de Sio and van den Hoven, the *designer* already plays a crucial role. The *tracing* condition, which also receives numerous mentions in the records we analysed,^9 15 16^ requires ‘[…] that there is at least one human agent in the design history or use context involved in designing, programming, operating and deploying the autonomous system […]’.^1^ In our sample, *designers* are mentioned as moral actors of MHC but also as those who enable MHC. For example, this could be by programming slow AI,^17^ adding certain validation and certification mechanisms to enable MHC,^4^ or developing a design for the implementation of MHC together with other actors in human-agent teams.^9^

Additionally, *legislative authorities* (n=6) and *researchers* (n=4) are often seen as enablers. There is a call for legislative projects similar to those for autonomous weapon systems or autonomous vehicles to be considered in order to regulate the use of automated systems in the health sector and achieve MHC. Kavidha and colleagues explain: ‘We must avoid the risk that robotics, AI and IoT [internet of things] be treated like oracles of previous times that humans unquestioningly relied upon to their detriment. […] Canadian health law and policy will eventually need to confront similar considerations for healthcare workers and their patients.’^18^ With regard to the European legal context, the extent to which the requirements of Article 22(3) of the General Data Protection Regulation (GDPR) can be taken into account in AI applications remains to be discussed.^19^

In addition, *researchers* (n=4) or entire research programmes can also enable MHC, and thus contribute to the controllability of AI application for society. One example is the research programme of TNO, the Netherlands Organisation for Applied Scientific Research.^20^

Finally, looking at the distribution of codes in the code group *enabler*, it is noticeable that the other codes have a very low frequency (see figure 2). However, the overall result for the code group shows that a variety of different actors can be involved in enabling MHC and how important a multiperson approach can be for implementing the concept of MHC in health.

#### Purposes of MHC

The vast majority of coded passages in the sample did not mention a purpose of MHC (n=124). For those that mention a purpose, the most common one was enabling *alignment with ethical or legal norms* (n=23). For example, failure to ensure MHC ‘[…] is considered a threat to human dignity, as it may open possible applications/decisions against humans or provoking harm to humans. This position is argued on the basis of the recognition of the principle of human dignity, in a human-centric approach, and principle of non maleficence (do no harm to humans) and beneficence (do good to humans) in bioethics.’^21^ In a similar vein, others propose as one dimension of MHC *‘[b]ehavioral compliance with moral values* […] which measures whether the [human-AI] team behavior corresponds with the human’s moral values.’^9^

The second most frequently mentioned purpose of MHC was the *attribution of responsibility* (n=22), including closely related notions such as answerability and accountability. Some authors even refer to exactly the same wordings to draw this connection (cf. 22 23): MHC means that ‘[…] humans not computers and their algorithms should ultimately remain in control of, and thus morally responsible for, relevant decisions […]’.^1^ As for the grounding of this connection, some highlight that ‘[t]he term MHC originated from the legal-political debate around lethal autonomous weapon systems […]. A serious concern driving this debate is the possibility of an accountability gap, where no one can be held accountable for potential war crimes committed by these systems.’^9^

#### Conditions of MHC

In addition to the actors who are supposed to enable MHC and the goals that are pursued with MHC, a third level is crucial: what are the conditions that are used as a basis for attributing MHC? In the sample, a wide range of conditions for attributing MHC can be found. The analysed records do not provide a consistent picture of which conditions are essential to the concept of MHC. Some conditions, however, are particularly notable. The conditions *tracing* (n=17) and *tracking* (n=12) as introduced by Santoni de Sio and van den Hoven^1^ are both mentioned frequently. Other scholars have taken up this idea and argue: ‘Thus, responsibility in healthcare drone design and operation can be reasonably assured if both conditions of MHC are met and incorporated into the system’s specifications.’^15^ The Global Initiative on Ethics of Autonomous and Intelligent Systems of the Institute of Electrical and Electronics Engineers (IEEE) supports this thesis, but specifies more precisely when it sees tracking and tracing fulfilled: ‘[…] we recommend that technical organizations promote a number of measures to help ensure that there is meaningful human control of weapons systems:

- That automated weapons have audit trails to help guarantee accountability and control.
- That adaptive and learning systems can explain their reasoning and decisions to human operators in transparent and understandable ways.
- That there be responsible human operators of autonomous systems who are clearly identifiable.
- That the behavior of autonomous functions should be predictable to their operators.
- That those creating these technologies understand the implications of their work.
- That professional ethical codes are developed to appropriately address the development of autonomous systems and autonomous systems intended to cause harm’^24^.

There is a consensus among the papers dealing with the conditions of MHC that *tracking* and *tracing* are central conditions for the attribution of MHC.

In addition, *informedness* (n=13) was the third most frequently mentioned condition. *Informedness* here is understood primarily with regard to the need for consent in the use and application of automated systems: ‘Robotic surgery involving increasingly autonomous systems inherits and extends ethical issues in RAS [Robot-Assisted Surgery] concerning the respect for patient autonomy and its application to informed consent procedures. Aspects of patient autonomy that must be carefully addressed in RAS are confidentiality maintenance and the adequacy of technological information provision. […] [O]ne must evaluate whether information disclosure must include selective information about the involved robotic systems and, if so, which amount of information is sufficiently rich and understandable for autonomous patient reflection and decision-making.’^25^ A particular difficulty is seen when ‘[…] an integral part of this information may concern the overriding privileges of human surgeons and their MHC powers more generally. Indeed, this information may prove crucial for patient proper evaluation and acceptance of risk arising from the use of autonomous surgical robots, especially in the early stages of their introduction, when reliable statistical projections about their future behaviours are not available yet.’^25^

### Enacting control

In a second step, we focus on how to understand this control. First, we address the question of who is in control. Second, we tackle the question of when and how control is meaningfully carried out. Third, we concentrate on what factors challenge these agents in exercising control.

#### Agents in control

Agents in control are those human actors and institutions that perform control in a meaningful way. The records often did not emphasise who exactly these agents are, but that the decision-makers are always *human* actors.^4^ In contrast to the enablers of MHC, only four distinct actors are tagged as agents in control. Particularly striking is the frequent mention of *clinicians* (n=25) as agents in control. By *clinicians* we mean all medical professionals. They are referred to in different areas of practice such as diagnosis of COVID-19,^3^ application of surgical robots,^25 26^ AI-based decision support systems in clinics,^4^ developing human-agent teams with a view of triage decision,^9^ regulating AI in health settings^21^ or in more general medical contexts.^18 27^ Depending on the field of practice, human control looks different and adopts a different mode. At the same time, it can also differ within a field of practice, as Fanny Ficuciello and her colleagues point out for the MHC of surgical robot autonomy: depending on the level of autonomy of the surgical robot, the human operator can carry out meaningful control in different ways, from ‘master-slave control mode’^25^ —in the case of a non-autonomous surgical robot—to monitoring and selecting only one of the strategies generated by the surgical robot (when the surgical robot is acting autonomously).^25^

One of the tasks of the human operators which robotic surgeons cannot accomplish is to prevent harm. Ficuciello and her colleagues show that human subjects have specific duties in contrast to robotic systems: ‘These duties must be sensibly distributed among involved human subjects, in accordance with their respective competences and professional roles–medical doctors, other members of medical staff and institutions, insurers, engineers, producers and designers of robotic equipment.’^25^ *Designers* (n=4) are one group of the agents in control. What exactly their duties are is not explained further. The focus is rather on the fact that they and also the other groups of agents can only manage these tasks together.

In some records, clinicians are mentioned together with *patients* (n=5). In their practice of MHC, *patients*, like physicians, must not be left alone.^21^ Rather, concrete legal frameworks are needed, as Kavidha and colleagues emphasise for patients and healthcare workers.^18^ The need for concrete models for patients’ MHC in addition to the legal framework is highlighted, because ‘[…] the ideal of meaningful control calls for concrete modes for individual control. Such modes of control could, for example, be implemented by envisioning patients as comanagers of their data and of the processes into which such information is channelled.’^4^ The question of control is closely linked to the assessment of what information patients need for their autonomous decision-making. With the help of the available information, patients can weigh up the risk (which cannot yet be estimated, especially in the initial phase due to the lack of survey data), and decide for themselves whether they should, for example, consent to surgery by a robot under human control.^25^

#### Time of determining MHC

Another aspect of MHC can be considered from a temporal perspective. The majority of text passages of the code group *time of control* left the temporal direction implicit, vague or *unspecified* (n=146). Some of the mentions were *forward-looking* (n=12) in the sense that MHC concerns future processes or outcomes. As one example, it was described as a forward-looking task to ‘[…] frame complementarity between man and machine, searching for ways of intelligent ‘support’ that allows man to have ‘significant or meaningful human control’ in terms of attention, contribution, supervision, control, and responsibility.’^23^ A further temporal aspect of MHC is to exert *continuous control* (n=8); for example, ‘[t]o ensure meaningful human control, operators should be able to query a system in real-time.’^24^ Finally, MHC plays a role in *retrospective* considerations in which the goal is to ascribe responsibility for past events (n=3). For such ascriptions, it can be taken as relevant whether or not there was MHC.^25^

#### Mode of MHC

A central question in the debates around MHC is what kind of control should be exercised over automated systems and in what form such control can be exercised. In the present sample, *exercising control* was the most frequently mentioned mode (n=29). Exercising control is understood as something that is specific to human agents in dealing with machine systems: ‘It is widely accepted that moral responsibility as an intrinsically human property cannot be allocated or shifted to algorithms or machines, however sophisticated they may be. AI systems exhibit ‘autonomy’ to some degree, in a sense that they are technically able to make predictions independently.’^19^ It is important to note that the rules around automated systems in healthcare are not yet clearly charted out: ‘In the current legal accountability system there is no provision for a non-human actor.’^28^

‘Hence, in order to uphold moral responsibility and accountability of humans the European Group on Ethics requires ‘meaningful human control’ being maintained and that humans ultimately remain in control of the decision-making process.’^28^ At the same time, the forms of control mentioned or those that are theoretically possible in each case must also be able to be actually implemented and achieve a control effect. As a condition for effectively (and in this sense meaningfully) exercised control, it is argued that human actors need sufficient time and must be able to sufficiently justify the reasons for exercising control. ‘The word ‘meaningful’ in MHC is meant to exclude control modes that one may nominally argue to incorporate humans in the control loop, even though human control is reduced there to a perfunctory validation of robotic actions, that is, in the absence of sufficient time and rationale to make an informed human judgment and to undertake the attendant actions.’^25^

Next to exercising control, *monitoring control* (n=19) was the second most discussed mode of control. Monitoring is essentially understood as analysing and evaluating the decision paths of automated systems: ‘For a decision based on data provided by a Covid-19 diagnosis app, meaningful review means that a human—ideally a healthcare professional—should be able to analyse the factors that led an application to a particular decision and, if needed, override them or refer the analysis to a human specialist.’^3^

#### Challenges of MHC

The agent in control is confronted with various challenges. As in other contexts where machine and deep learning algorithms are used, *algorithmic opacity* (n=8) is one issue: ‘The difficulty is exacerbated by the fact that self-learning robots often operate in ways that are opaque to humans, even their programmers’.^29^ Other main challenges of MHC include the question of how to *process inefficiencies through human supervisors* (n=5), as well as the challenge to maintain *transparency* (n=5).

### Evaluating control

The last field of action draws attention to the evaluation of MHC: who carries out this evaluation process and who should do it? How is the concept of MHC generally perceived and evaluated in the research debate?

#### Evaluators of MHC

Evaluators are understood as human actors or institutions that examine whether or not there is or has been MHC. Christine Harvey and her colleagues describe the act of evaluating MHC as challenging: ‘Maintaining meaningful human control is essential, but difficult to translate into requirements and evaluate in completed systems.’^30^

Fanny Ficuciello and her colleagues show for the case of surgical robots that, for example, in liability issues, MHC obligations need to be evaluated in relation to the responsibilities of the surgeon. However, they do not address the question of who should take on this evaluation task.^25^

*Executive authority* was coded as evaluators of control. *Executive authority* (n=2) was mentioned in two contexts. First, in connection with autonomous weapon systems in the question of whether MHC is used in critical decisions.^31^ Second, in discussions of the extent to which MHC can be ensured through legal regulation in all automated systems, despite an unclearness in law and slowness of governmental processes.^30^ In this case, *executive authority* can be considered to be an enabler as well as an evaluator.

#### Critique of MHC as normative guiding principle

Most passages left their own stance on the suitability of MHC as a normative guiding principle *unspecified* (n=103) and mentioned MHC in primarily descriptive, non-evaluative statements, for example, when stating that with automated AI, ‘[t]here is also an issue of meaningful human control (MHC). The question is why and to what extent human control in AI is necessary or desirable for decision making in certain contexts.’^32^ Other passages were more unequivocally *affirmative* (n=59) when referring to MHC as a guiding principle: for example, when they explicitly ‘[…] recommend that technical organizations promote a number of measures to help ensure that there is meaningful human control of weapons systems’.^24^ Lastly, a smaller number of passages were *critical* (n=6) of MHC as a requirement for the use of AI, for example, when arguing ‘[…] that one may have to give up MHC in some emergency situations, on account of medical beneficence considerations, enabling robots to act with unconditional control capabilities in task execution.’^25^ Some degree of scepticism about MHC arose from the perceived vagueness of the concept, for example, when reporting that some ‘[…] states have focused on artificial intelligence, robot armies, or whether ‘meaningful human control’–an undefined term–is exercised over life-and-death decisions.’^31^

## Discussion

Although the two conditions *tracking* and *tracing* are unanimously seen as central conditions for the attribution of MHC, they are mentioned in only 8 of the 42 records. Some records^9 15 16^ even cite the definition of Santoni de Sio and van den Hoven,^1^ while another record applies the conditions to a health setting.^3^ The full content of these conditions is not always illuminated. For example, references to the *tracing* condition do not always make explicit that tracing also involves epistemic conditions, given that part of the formulation of *tracing* is that ‘[…] for a system to be under meaningful human control, its actions/states should be traceable to *a proper moral understanding on the part of one or more relevant human persons* who design or interact with the system […]’^1^ (our emphasis). There is a need for further elaboration on what such understanding encompasses, especially with regard to the practical implementation of MHC in different health settings. Recent publications address this difficulty and propose further cornerstones to help implement the concept from theoretical considerations into practice.^33^

The discussion about MHC in the health sector, which started in 2016, is still in its infancy. The high number of *unspecified* codes in the individual code groups within our results shows that the concept is sometimes only treated superficially. The individual components of MHC have been researched and discussed to very different degrees. Our sample reveals little about who is assigned which task in the control process, and how MHC is actually understood and operationalised. The reflections of Ficuciello and colleagues or Jasper van der Waa and colleagues are rather the exception here.^9 25^

One question runs like a thread through the results we present: how are responsibility and MHC related? On one standard view, MHC over relevant AI-driven clinical processes is a necessary condition for clinicians’ responsibility for the particular outcome. In short, there can be moral responsibility only if there was MHC.^34^ This resonates with the above-mentioned *purpose* of MHC to prevent responsibility gaps.^1^ Based on our review, we can point to an additional, explanatory direction which takes responsibility as prior: clinicians have a responsibility to ensure that there is MHC over AI-driven clinical processes. ‘Indeed, a surgeon might be held responsible for damages caused by an autonomously performing robot if she failed to exert MHC properly […]’.^25^ On the other hand, if there is or was MHC, this can exempt the agent from responsibility attributions as they ‘[…] might be correctly rebutted […] by showing that the specified MHC duties were carefully complied with.’^25^ What this suggests is that the bundle of responsibilities of the clinician operating with AI-driven tools is multifaceted and nested with MHC in different ways. Responsibility for outcomes might presuppose that there is MHC, but even before considering such concrete outcomes, our review suggests that a broader responsibility of the clinician and presumably further stakeholders is to prevent situations in which MHC is lacking in the first place.

For this, other stakeholders must also be considered. The records we analysed are often limited to designers being the sole enablers of MHC and clinicians being the controllers. But what other actors or institutions can also exercise control? Our take on the concept of MHC in health suggests that moving MHC away from the often sole focus on designers or clinicians (the most common codes in the enabler and controller groups) is warranted. As Luciano Cavalcante Siebert and colleagues note: ‘Meaningful human control refers not only to the development of the AI agent, but also to the design of the sociotechnical environment that surrounds it, including social and institutional practices’.^33^ MHC is applied in specific, often pre-existing situations (eg, surgery or medical diagnosis) that shape the concept of MHC. Since MHC is influenced and shaped by this particular sociotechnical environment, the first question is which institutions and actors need to work together to make MHC possible in different health settings in the first place. Second, how should those whose health or viability is at stake, namely the patients, be involved in the concept of MHC? To what extent could patients and relatives have more controlling functions here, or could this task also be taken over more by society? Are there special skills that the actors in control should need? And third, how should the evaluators be able to give feedback on their findings to the other two groups of actors?

**Figure 3:**
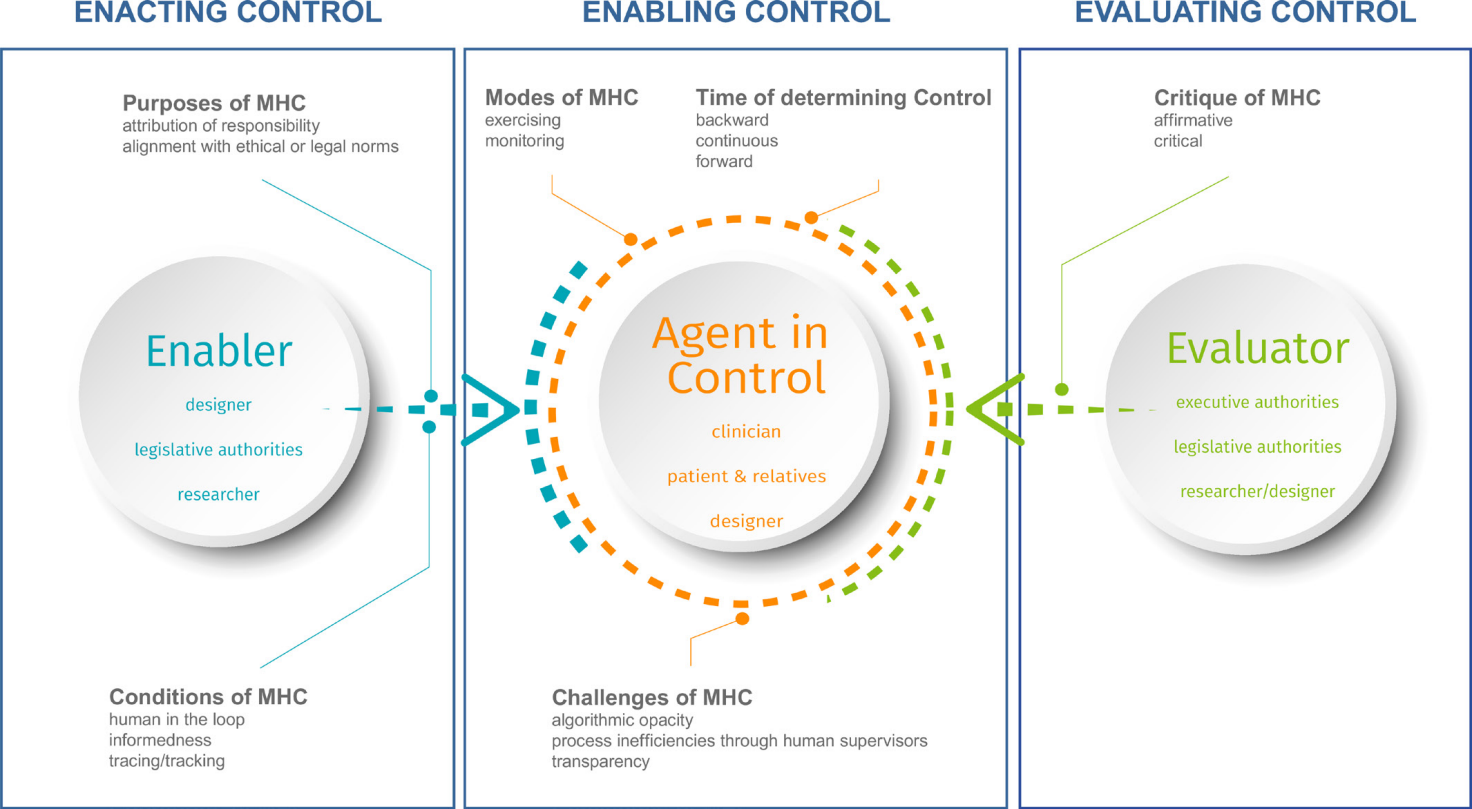
A broader take on the concept of MHC in health. MHC, meaningful human control.

A more systematic approach to MHC is required to address these questions and to advance the discussion of the concept of MHC in health, both to better understand it and to put it into practice. In order to guide implementation and operationalisation, such an approach must move the concept towards greater attention to all the actors involved, and, second, it must highlight the complexity of the areas in which it is used: different environments shaped not only by technical, but also by social and legal anchors. We therefore propose examining MHC in health along three different strands of action: enabling, enacting and evaluating control (figure 3). On the one hand, the schema is created inductively from the data material; on the other hand, we find that the data material illuminates different components of the schema only superficially. The three strands recall the familiar stages of policy planning, implementing and evaluating. It should be noted, however, that, unlike some other processes, the three strands themselves are not to be worked through sequentially, but represent an iterative process. The focus on three strands makes it possible to facilitate the implementation and operationalisation of MHC in practice, while taking into account the complexity of (implementing) automated systems in health settings. In addition, the different actors are central to this model (those most frequently mentioned in our database research are shown in the circles). The purpose of the proposed take is twofold: first, to accurately capture the concept of MHC with all its challenges and complexities in different health settings. Second, to make it easily adaptable to different settings in the health sector where automated systems are implemented in, as well as to non-health settings where automated systems could also play a decisive role.

This approach should help to bridge the gap between the theoretical concept of MHC and its implementation in health. However, when analysing the different conceptions and understandings of MHC, two central gaps emerge that need to be kept in mind: the theoretical gap and the labelling gap.

First, MHC is still an open concept that needs interpretation. This is somewhat in tension with the perception of MHC as a clearly elaborated control concept. While this may be more accurate with regard to the use of MHC in the context of automated weapons systems, it is not the case with regard to MHC in the context of health. Two central reasons can be found for this. The first reason is that the normative questions that arise—especially when trying to determine when a form of control is ‘meaningful’—seem to be even more diverse and complex than in the context of autonomous weapon systems. A second reason could be the significant decision-making constellations in the health sector, which involve many different actors and diverse moral and legal reasons and claims. It is good and important that many concepts of MHC refer to ethical principles and criteria. However, this reference remains relatively vague as long as it is not integrated into a corresponding framework in which the connection between the specific context of application, the relevant moral intuitions and values, and the possible ethical and legal points of orientation is clarified.^4 35^ The concrete orientation that each concept of MHC can provide for the particular context of application thus depends crucially on the clarification of the corresponding conceptual reference point for MHC. This currently represents a research gap with regard to MHC, which we suggest referring to as the theoretical gap.

Second, in current debates on MHC in the context of health, a tendency can be observed to consider MHC as a kind of label for a certain moral or legal virtue of the automated system.^36^ On the one hand, such efforts can be very helpful in discussing points of intervention for control, which are then taken into account in the design of the systems themselves. However, at the same time, there is a risk of losing sight of the fact that the question of meaningful control, especially with regard to the health sector, cannot be separated from the institutional framework conditions that surround it, nor from broader moral concepts. The latter can be illustrated subsequently to our thoughts above with regard to questions of responsibility, since the question of attributing responsibility is of central importance, especially in the context of medical decision-making. Let us assume that with regard to a system, MHC can be attributed. The question of whether it is also responsible to apply this system in a specific context cannot yet be answered. It is therefore conceivable that MHC exists, and yet it is irresponsible to apply this system. MHC and the attribution of responsibility must thus be regarded as different forms of evaluation. We think that there are certainly good reasons to argue that MHC could be a condition, possibly even a necessary condition, for the attribution of responsibility in the use of an automated system. But the attribution of meaningful control cannot replace an ethical assessment and likewise a social debate about responsible use. Otherwise, there is a risk of what we call a labelling gap—that the use or implementation of an automated system will be morally or legally justified by the label of MHC itself, without consideration of whether the application is actually ethically justifiable in terms of the institutional frameworks and moral conceptions involved.

At this point, it is important to return to the first point: the use and application of automated systems in medical contexts take place in specific contexts for which there are already established rules and procedures.^37^ For example, if an automated system is used for a specific part of medical decision-making, there are already institutionally established procedures for this decision-making, harm mitigation bodies,^10^ rules for liability in decision-making and, last but not least, a medical ethics framework.^38^ The crucial point here is that the existing institutional framework settings already define the minimum requirements for control that different actors such as clinicians, patients, caregivers, relatives and others can claim as entitlements and rights that have already been conceded. What kind of control is meaningful is therefore not just solely a technical question, but a social one.

## Conclusion

This review examines the current state of the concept of MHC in the context of health. In this field, human actors are particularly needed for harm reduction when using automated systems. As the findings show, to date, there is no robust MHC concept for health. Therefore, we propose a broader understanding of MHC that is oriented towards the aspects of enabling, enacting and evaluating control. The presented take on MHC provides an opportunity to systematically address the use of automated systems in the different health sectors in three steps: first of all, the designers of MHC as well as legislative authorities, researchers and others (see figure 2) need to determine the purpose of MHC and the conditions for its use. Second, the concrete implementation of MHC requires clarification of who is involved in it (clinicians, patients and representatives, designers, society or others). Furthermore, the mode and time at which the control is established and potential challenges in the implementation need to be analysed. Third, the stakeholders of the evaluation need to verify whether MHC is ensured and whether the defined purpose of MHC has been fulfilled. These strands flow into each other as iterative processes. By considering these different aspects, the MHC approach can prove to be a robust framework that serves its purpose under a wide variety of conditions, taking into account the complexity of the area in which it is implemented and the individual characteristics of the environment. This can be achieved by being able to respond directly to unexpected failures, by setting up in advance a system of actors who think about failure mechanisms from the perspective of enabling, implementing and evaluating control, and who anticipate possible failures and jointly develop ways of dealing with them.

Taking into account these strands of action and the established rules and processes in different health sectors, the MHC concept needs further development to avoid falling into two potential gaps, which we have described as theoretical and labelling gaps.

To avoid the theoretical gap, a closer look at the different stakeholders and further discussion of initial trends in research are needed, such as the importance of a multiperson approach for the implementation of the concept of MHC in different health settings.

For the labelling gap, it is crucial to not only use MHC as a label but to take into account the corresponding institutional framework and values, too. Much more attention must also be paid to the specifics of health: first, the individual preferences of those affected. For example, patients, relatives or medical staff may exclude treatment options based on their values and perceptions. Second, the individual need for control. How do people differ in their need for control? How can this need be satisfied? When is the control exercised meaningful? Third, the relevant conceptual reference points. All these aspects need to be taken more into account if MHC is not just to remain a label in health.

## Supplementary material

10.1136/jme-2023-109095 online supplemental file 1

## Data Availability

All data relevant to the study are included in the article or uploaded as supplemental information.

## Footnotes

Since wildcards are not allowed in ScienceDirect and Web of Science databases, the search strategy was changed as follows: (health OR healthy OR medicine OR medical OR clinic OR clinical OR clinician) AND “meaningful human control”.

If the record did not have an abstract, we analysed the summary; if the summary was not available, we analysed the introduction.

The excluded publications were mainly dedicated to the following contexts: ethics of AI; happiness; industry 4.0/5.0; law (commercial law, international humanitarian law); military (autonomous weapon systems; The North Atlantic Treaty Organization (NATO)); transport (autonomous, self-driving vehicles); policy analytics and societal impact.

This step excluded many records that only mentioned the article of Fanny Ficuciello and her colleagues^25^ in their bibliography. In the search databases, many records were therefore displayed as results, although MHC was not mentioned in the articles themselves.

As with the first search strategy, health relevance was indicated by “health*”, “medic*” or “clinic*” or other direct references to a health context.

In cases of MHC being mentioned in bulleted paragraphs, the relevant bullet point was analysed.

In order to exclude the possibility that the codes were chosen incorrectly and therefore cannot represent the content, the *unspecified* codes of the nine code groups were checked again for the addition of further codes.
