# Supplementary material for: Meaningful Human Control over AI for Health? A Review
Source: J Med Ethics. 2023 Sep 20;52(e1):e109095. doi: 10.1136/jme-2023-109095 (PMC13151516; doi:10.1136/jme-2023-109095)
Supplement: online supplemental file 1 [file jme-52-e1-s001.pdf]

Almada, M. and J. Maranhão (2021). "Voice-based diagnosis of covid-19: ethical and legal challenges." International data privacy law.

Assadullah, M. M. (2019). Barriers to Artificial Intelligence Adoption in Healthcare Management: A Systematic Review, University of Maryland Global Campus.

Braun, M., et al. (2021). "A Leap of Faith: Is There a Formula for “Trustworthy” AI?" Hastings Center Report.

Braun, M., et al. (2020). "Primer on an ethics of AI-based decision support systems in the clinic." J. Med. Ethics.

Brey, P., et al. (2019). D3.2 Guidelines for the development and use of SIS, Shaping the ethical dimensions of smart information systems – a European perspective (SHERPA).

Cawthorne, D. and A. Robbins-van Wynsberghe (2020). "An ethical framework for the design, development, implementation, and assessment of drones used in public healthcare." Sci. Eng. Ethics 26(5): 2867-2891.

COMEST (2017). Report of Comest on Robotics Ethics. COMEST Reports. Paris, UNESCO.

Connor, M. J., et al. (2020). "Autonomous surgery in the era of robotic urology: friend or foe of the future surgeon?" Nature Reviews Urology 17(11): 643-649.

Crawford, K., et al. (2016). The AI Now Report. The Social and Economic Implications of Artificial Intelligence
Technologies in the Near-Term. A summary of the AI Now public symposium, hosted by the White House and New York University’s Information Law Institute, July 7th, 2016. AI Now.

David, W. and M. King-Okoye (2020). "Artificial Intelligence and Robotics Addressing COVID-19 Pandemic’s Challenges." International Conference on Modelling and Simulation for Autonomous Systems: 279-293.

Ficuciello, F., et al. (2019). "Autonomy in surgical robots and its meaningful human control." Paladyn, J. Behav. Robot. 10(1): 30-43.

Fosch-Villaronga, E. and H. Drukarch (2021). On Healthcare Robots. Concepts, definitions, and considerations for healthcare robot governance. arXiv preprint arXiv:2106.03468, eLaw Center for Law and Digital Technologies
Leiden University.

Gupta, A., et al. (2021). The State of AI Ethics Report (Volume 5), Montreal AI Ethics Institute.

Harvey, C., et al. (2017). December 2016. Federal Big Data Summit Report, The Mitre Corporation.

Hawley, S. H. (2019). Theopolis Monk: Envisioning a Future of AI Public Service. The Transhumanism Handbook, Springer: 271-300.

Hummel, P. and M. Braun (2020). "Just data? Solidarity and justice in data-driven medicine." Life Sci. Soc. Policy 16(1): 1-18.

Jacobs, F. (2018). Safety through Machine Learning Applications. A Safety Case Analysis, TU Delft.

Johnson, M., et al. (2021). "Responsible Artificial Intelligence in Healthcare: Predicting and Preventing Insurance Claim Denials for Economic and Social Wellbeing." Information Systems Frontiers: 1-17.

Kavidha, V., et al. (2021). "AI, IoT and Robotics in the Medical and Healthcare Field." AI and IoT-Based Intelligent Automation in Robotics: 165-187.

Kowalczewska, K. (2019). "The Role of the Ethical Underpinnings of International Humanitarian Law in the Age of Lethal Autonomous Weapons Systems." Polish Political Science Yearbook 48(3): 464-475.

National Science and Technology Council (2016). Preparing for the future of Artificial Intelligence Washington D.C., Executive Office of the President of the United States.

Palazzani, L. (2021). "AI and health: ethical aspects for regulation." Teoria e Critica della Regolazione Sociale/Theory and Criticism of Social Regulation.

Puaschunder, J. M. The legal and international situation of AI, robotics and big data with attention to healthcare.

Rehn, A. (2020). Dylan Cawthorne. Value Sensitive Design of Unmanned Aerial Systems, Maersk Institute. University of Southern Denmark.

Rejali, S. and Y. Heiniger (2020). "The role of digital technologies in humanitarian law, policy and action: Charting a path forward." International Review of the Red Cross 102(913): 1-22.

Roff, H. M. (2019). "Artificial intelligence: Power to the people." Ethics & International Affairs 33(2): 127-140.

Roumate, F. (2020). "Mechanisms of Advanced International Psychological Security in the Age of Artificial Intelligence." Коммуникации. Медиа. Дизайн 5(1): 143-156.

Schönberger, D. (2019). "Artificial intelligence in healthcare: A critical analysis of the legal and ethical implications." Int. J. Law Inf. Technol. 27(2): 171-203.

Sipior, J. C. (2020). "Considerations for development and use of AI in response to COVID-19." International Journal of Information Management 55.

Stephanidis, C., et al. (2021). "Human factors in ambient intelligence environments." Handbook of Human Factors and Ergonomics: 1058-1084.

Stephanidis, C., et al. (2019). "Seven HCI Grand Challenges." INT J HUM-COMPUT INT 35(14): 1229-1269.

Sundaraj, N. D. (2020). "The Curious Case of Doctor Ultron: How Well Is English Law Currently Suited to Manage the Inherent Risks Associated with Black Box AI Medical Diagnostics?" Cambridge L. Rev. 5: 58.

Szpak, A. (2020). "Legality of Use and Challenges of New Technologies in Warfare–the Use of Autonomous Weapons in Contemporary or Future Wars." European Review 28(1): 118-131.

The IEEE Global Initiative on Ethics of Autonomous and Intelligent Systems (2017). Ethically aligned design: A Vision for Prioritizing Human Well-being with Autonomous and Intelligent Systems. Version 2, IEEE.

Vallor, S. and G. A. Bekey (2017). "Artificial intelligence and the ethics of self-learning robots." Robot Ethics 2.0: 338–353.

van der Waa, J., et al. (2020). "Allocation of moral decision-making in human-agent teams: a pattern approach." Computer Science 12187: 203-220.

van der Waa, J., et al. (2021). "Moral decision making in human-agent teams: Human control and the role of explanations." Front. Robot. AI 8.

van Est, R., et al. (2017). Human rights in the robot age. Challenges arising from the use of robotics, artificial intelligence, and virtual and augmented reality, Rathenau Instituut.

van Stijn, J. (2021). "Moral Decision-Making in Medical Hybrid Intelligent Systems: A Team Design Patterns Approach to the Bias Mitigation and Data Sharing Design Problems."

Werkhoven, P. J. (2018). TNO Early Research Program Annual plan 2019, TNO.

Zaman, M. F. and N. Buchholz (2021). "Robotic Surgery and Its Application in Urology: A Journey Through Time." UROLOGY.

Zicari, R. V., et al. (2021). "On Assessing Trustworthy AI in Healthcare. Machine Learning as a Supportive Tool to Recognize Cardiac Arrest in Emergency Calls." Front. Hum. Dyn. 3: 30.
